# Cultural and linguistic transferability of the multi-dimensional OxCAP-MH capability instrument for outcome measurement in mental health: the German language version

**DOI:** 10.1186/s12888-018-1762-3

**Published:** 2018-06-05

**Authors:** Judit Simon, Agata Łaszewska, Eva Leutner, Georg Spiel, David Churchman, Susanne Mayer

**Affiliations:** 1 Department of Health Economics, Center for Public Health, Medical University of Vienna, Kinderspitalgasse 15/1, 1090 Vienna, Austria; 2 Department of Psychiatry, University of Oxford, Warneford Hospital, Oxford, OX3 7JX UK; 3 Health Economics Research Centre, Nuffield Department of Population Health, University of Oxford, Richard Doll Building, Old Road Campus, Oxford, OX3 7LF UK; 4 pro mente Kärnten, GmbH, Villacherstraße 161, 9020 Klagenfurt, Austria; 5 pro mente Forschung, Villacherstraße 161, 9020 Klagenfurt, Austria; 6 Oxford University Innovation (Clinical Outcomes), Buxton Court, 3 West Way, Oxford, OX2 0JB UK

**Keywords:** Mental health, Quality of life, Capabilities, PROM, Translation, Validation

## Abstract

**Background:**

Mental health conditions affect aspects of people’s lives that are often not captured in common health-related outcome measures. The OxCAP-MH self-reported, quality of life questionnaire based on Sen’s capability approach was developed in the UK to overcome these limitations. The aim of this study was to develop a linguistically and culturally valid German version of the questionnaire.

**Methods:**

Following forward and back translations, the wording underwent cultural and linguistic validation with input from a sample of 12 native German speaking mental health patients in Austria in 2015. Qualitative feedback from patients and carers was obtained via interviews and focus group meetings. Feedback from mental health researchers from Germany was incorporated to account for cross-country differences.

**Results:**

No significant item modifications were necessary. However, changes due to ambiguous wordings, possibilities for differential interpretations, politically unacceptable expressions, cross-country language differences and differences in political and social systems, were needed. The study confirmed that all questions are relevant and understandable for people with mental health conditions in a German speaking setting and transferability of the questionnaire from English to German speaking countries is feasible.

**Conclusions:**

Professional translation is necessary for the linguistic accuracy of different language versions of patient-reported outcome measures but does not guarantee linguistic and cultural validity and cross-country transferability. Additional context-specific piloting is essential. The time and resources needed to achieve valid multi-lingual versions should not be underestimated. Further research is ongoing to confirm the psychometric properties of the German version.

**Electronic supplementary material:**

The online version of this article (10.1186/s12888-018-1762-3) contains supplementary material, which is available to authorized users.

## Background

Mental health conditions can have a substantial effect on quality of life, well-being and physical and social functioning [1, 2]. People suffering from mental health problems often face social stigma and experience discrimination or social isolation. Connel et al. (2014) found that aspects perceived by mentally ill people as the most important concerning their quality of life include quality of relationships, sense of belonging and acceptance, self-perception, autonomy and freedom of choice and feeling of hope [3]. These important aspects of well-being are often overlooked in the currently existing generic patient self-reported quality of life outcome measures (PROMs) [4, 5]. For example, the focus of the most commonly used preference-based measure in economic evaluations, the EuroQoL EQ-5D, lies mostly on different dimensions of physical health. It covers five dimensions of quality of life: mobility, self-care, usual activities, pain/discomfort and anxiety/depression which are expressed in three or five levels of severity [6, 7]. In recent years, concerns have been raised whether the EQ-5D is an accurate outcome measure in mental health research [4]. The EQ-5D does not always detect the change in quality of life in cases of severe mental health problems such as bipolar disorder [8]. Saarni and colleagues (2010) found that existing self-reported quality of life questionnaires, EQ-5D in particular, are not sensitive enough and do not always correlate with the clinical outcomes or socio-economic situation of patients [8].

In light of the ongoing discussion on the appropriateness of the existing outcome measures in psychiatric care, a new concept of measuring the quality of life/well-being in mental health research has been developed [9, 10]. The Oxford CAPabilities questionnaire-Mental Health (OxCAP-MH) is a novel 16-item index measure. Its theoretical background lies in Sen’s capability approach [11] and its later interpretation by Nussbaum [12]. According to Sen’s theory, the term “capabilities” refers to the “alternative combinations of things a person is able to do or be – the various ‘functionings’ he or she can achieve” [13]. The capability approach focuses on a person’s freedom to choose to do things that they value in their life. It can refer to very basic aspects, such as being well nourished or sheltered, and to more complex concepts such as social integration or self-respect [13]. The scope of the OxCAP-MH to capture quality of life is broad and multi-faceted and includes complex aspects of individuals’ well-being such as social integration, exposure to discrimination and social stigma. In particular, the OxCAP-MH refers to capability domains including: daily activities; social networks; losing sleep over worry; enjoying social and recreational activities; having suitable accommodation; feeling safe; likelihood of discrimination and assault; influencing local decisions; freedom of expression; appreciation of nature; respecting and valuing people; friendship and support; self-determination; imagination and creativity and access to interesting activities.

The capability approach has been increasingly applied in general outcome measurement in health economics. Instruments including ICECAP (ICEpop CAPability) [14, 15] and ASCOT (Adult Social Care Outcome Toolkit) [16] have been developed to use in the health and social care sectors [17]. The application of the OxCAP-MH currently remains in the area of mental health research. The instrument was used for the first time and validated in a randomised controlled trial in which study participants suffered from severe mental health conditions [9, 18]. The analysis showed that the instrument significantly correlated with social functioning (Global Assessment of Functioning, GAF) and health-related quality of life (EQ-5D VAS and EQ-5D-3L). A response rate above 70% and positive patient feedback indicated good feasibility of the instrument [9, 18]. These results suggest that the OxCAP-MH could be a suitable alternative for quality of life/well-being measurement in mental health research. However, more research is needed to examine its cross-cultural application and inter-country transferability to different linguistic and cultural settings since, until now, the instrument has been available only in English. The main objective of this study was to develop a linguistically and culturally valid German version of the OxCAP-MH to facilitate its use in German speaking countries and international research. We describe the complete translation and linguistic and cultural validation processes of the questionnaire and discuss any difficulties and queries that arose throughout this process and can serve as a guide to other similar adaptation processes.

## Methods

**Fig. 1:**
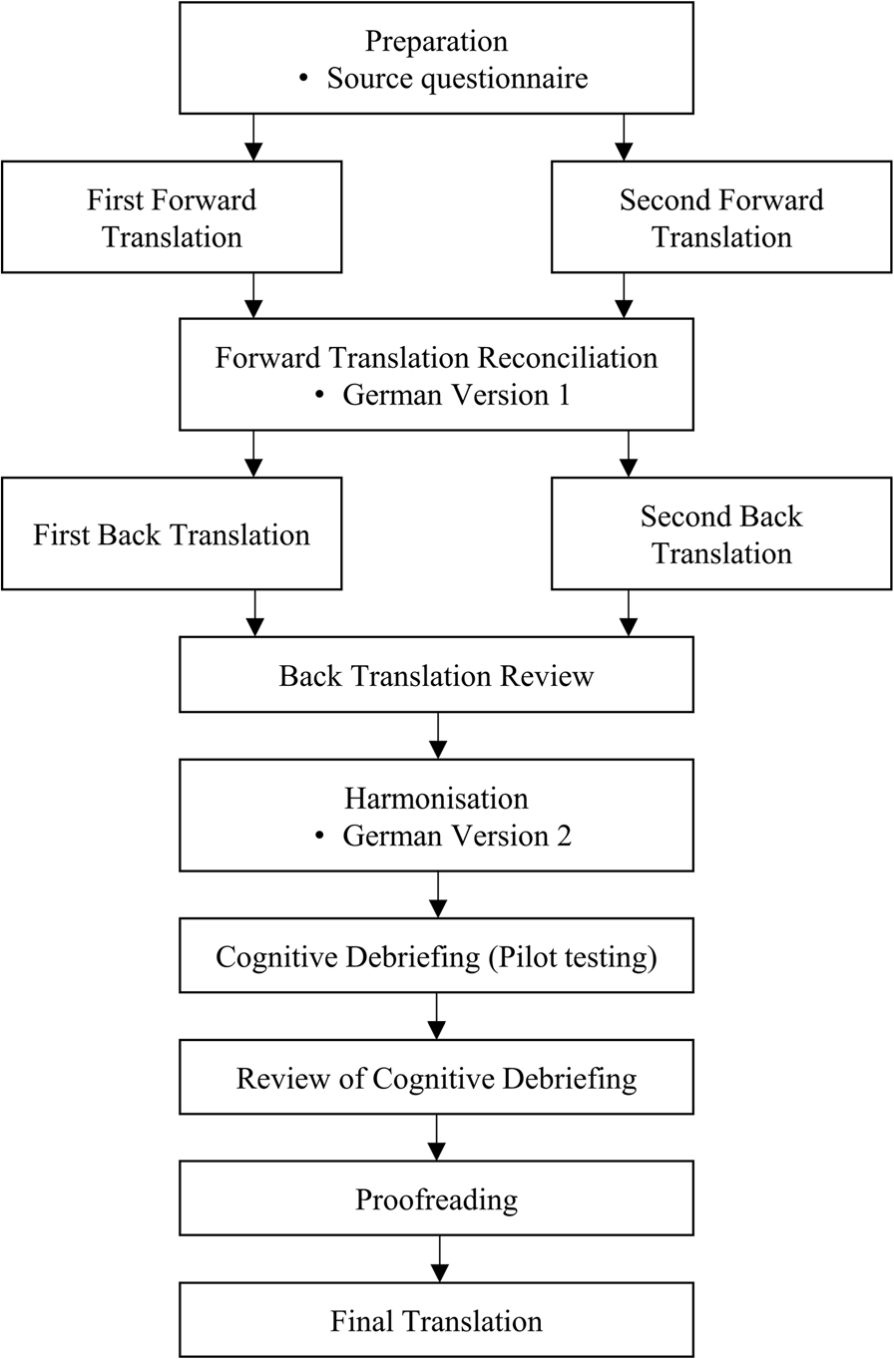
Translation process of OxCAP-MH according to PharmaQuest’s standard translation and ISPOR guideline

The professional translation and linguistic validation process was coordinated by the Clinical Outcomes team at Oxford University Innovation (previously Isis Innovation Ltd.), University of Oxford, UK and was carried out by PharmaQuest Ltd., a company specialising in the translation and linguistic validation of patient-reported outcome measures (PROMs). The quality of this process was ensured by complying with international principles of good practice for PROMs’ translation according to the International Society for Pharmacoeconomics and Outcomes Research’s (ISPOR) standards [19] and following relevant guidelines [20]. The process of German translation and linguistic validation of the OxCAP-MH is outlined in Fig. 1. To strengthen the conceptual equivalence of the target questionnaire to the original English version, instrument developers and an in-country investigator based in Austria were involved in the translation process. Communication between them and project investigators and qualified translators was maintained throughout the whole project period (March 2015–August 2015).

### Forward translation

The forward translation from English to German language was carried out by two independent qualified translators, native German speakers, who were proficient in English, specialised in medical translations and had a minimum of three years of experience. Two independent German versions of the questionnaire were produced, on which a reconciled single version was created (Version 1).

### Back translation and back translation review

The reconciled version of the German language instrument (Version 1) was back translated into English by two independently working translators who were English native speakers with proficiency in German. The in-country investigator commented on any queries regarding wording and terminology that arose from the back translations. In addition, two mental health specialists from Hamburg University of Applied Sciences, Germany provided further feedback and information on this preliminary version of the German OxCAP-MH questionnaire. The complete back translation review process allowed exploration and finding of equivalent translations to all questionable items. The amended version (Version 2) of the German questionnaire could then be pilot tested on a sample of patients with mental health conditions.

### Cognitive debriefing (pilot testing)

The aim of the pilot testing was to confirm whether the translations were accurately understood against the intended meaning of the original (English) OxCAP-MH questionnaire. The pilot test drew on a sample of 12 study participants who were the patients of “pro mente Kärnten GmbH”. Pro mente is an organisation that provides care and support for patients with mental health conditions in their everyday life in different organisational settings including crisis services, mobile care and assistance, psychosocial counselling, day centres and housing. It is located in the Austrian province Carinthia.

The study participants were approached by the carers in the respective institutionalised settings. The relevant selection and inclusion criteria for the patients were as follows: being able and willing to give written consent, aged between 18 and 65 years old, native German speaker, and not in an active phase of their mental condition. All participants received an oral and written information on the study and were asked to give an informed written consent prior to the face-to-face interview.

The carers were trained by the in-country investigator to carry out the interviews in accordance with the guideline provided by Oxford University Innovation [20]. Firstly, the patients were asked to complete the translated questionnaire alone. Secondly, the questionnaire was read aloud by the carers while the patients could follow it by having the questionnaire in front of them. Thirdly, the study participants were asked to comment on any wording that was difficult for them to understand and if applicable, suggest alternative wording. Patients were asked to describe in their own words what the wording meant to them. All interviews were recorded, transcribed and translated into English and analysed qualitatively using a content analysis approach [21].

In addition, four care workers and interviewers within pro mente Kärnten provided further feedback and information on Version 2 of the German OxCAP-MH questionnaire in a focused group discussion setting. The group indicated some additional difficult or odd wordings and gave suggestions of alternative wording of the items.

The pilot study was approved by the Ethics Committee of the Medical University of Vienna (EK-No.: 1900/2014 Votum 27.03.2015).

## Results

### Translation

Twenty-nine phrases were translated from the English source questionnaire to German, of which sixteen phrases corresponded to the questions comprising the final OxCAP-MH instrument. Two phrases were additional questions not included in the final score, four were instructions (e.g. *“Please tick one”*), six were different response options, and one was an explanatory sentence included at the beginning of the questionnaire, i.e. *“This questionnaire asks about your overall quality of life.”*

Following the formal steps of the translation process (Fig. 1), three German versions of the questionnaire were developed with the third version being the final approved translation of the OxCAP-MH. First, two independent forward translations were conducted and Version 1 of the German questionnaire was created. After conducting two independent back translations of Version 1 and carrying out the back translation review, nine out of 29 phrases (31%) were changed (seven based on the suggestions of the professional translators and two based on feedback from the in-country investigator in Austria and mental health specialists from Germany) and Version 2 was developed (Table 1).

**Table 1:** Change analysis

		Back translation review			Cognitive debriefing			Overall change (Yes/No)
Phrase	Content	Reason	Source	Accepted	Reason	Source	Accepted	
I0	Explanation	L	2	+	L	2	+	N
Q1	Daily activities				I	3	+	Y
I1	Instruction 1							
R1–4	Response option 1	I	1	+				Y
Q2	Social networks							
Q3	Losing sleep	I	1	+				Y
Q4	Enjoying recreation							
Q5	Suitable accommodation							
R5	Response option 2							
Q6	Neighbourhood safety							
R6	Response option 3							
Q7	Potential for assault				I	2	–	N
R7–8	Response option 4							
Q8	Discrimination							
I2	Instruction 2	I	1	+				Y
Q8a	Additional question	I	1	+				Y
I3	Instruction 3							
R8a	Response option 5				P	2, 3	+	Y
Q9	Additional question							
I4	Instruction 4				I	2	+	Y
R9	Response option 6	I	1	+				Y
Q9a	Influencing local decisions	L	2	+	S	2	–	Y
Q9b	Freedom of expression							
Q9c	Appreciating nature							
Q9d	Respect and appreciation	I	1	+				Y
Q9e	Love and support				I	3	+	Y
Q9f	Planning one’s life							
Q9g	Imagination and creativity							
Q9h	Access	I	1	+	I	2	–	Y
Number of accepted changes (%)		9 (31%)			5 (17%)			12 (41%)

Phrase: I = instruction, Q = question, R = response option

Reason: S = differences in political and social systems, L = cross-country language differences, I = possibilities for differential interpretation, P = politically unacceptable expressions

Source: 1 = professional translators; 2 = in-country investigator and/or mental health specialists; 3 = patients and/or carers

Overall change: + = suggested change accepted; − = suggested change rejected

### Cognitive debriefing

Eight women and four men participated in the pilot testing of the German OxCAP-MH questionnaire (Version 2). The mean age of study participants was 37 years (range 24–62 years). The most common diagnosis was depression (*n* = 5). The average duration of the interviews including both the times for completion and cognitive debriefing was 16 min (range 5–40 min) (Table 2).

**Table 2:** Cognitive debriefing (pilot study) sample characteristics

Patient ID	Age	Sex	Time (min.)	Diagnosis
001	24	Male	7	Schizophrenia
002	28	Male	10	Paranoid schizophrenia
003	36	Female	9	Depression, panic disorder
004	34	Female	10	Depression, panic disorder
005	26	Female	11	Borderline personality disorder
006	27	Female	5	Depression, panic disorder
007	32	Male	6	Depression, mental and behavioural disorders due to use of alcohol
008	62	Female	26	Schizoaffective disorder
009	50	Male	30	Depression, anxiety disorder
010	31	Female	23	Posttraumatic stress disorder
011	50	Female	20	Bipolar disorder
012	41	Female	40	Schizoaffective disorder

As the cognitive debriefing sessions revealed, neither patients nor carers experienced any major difficulties with understanding the individual item concepts or answering them. For patients, nine (56%) out of the 16 questions were entirely clear and six questions were easy to understand. Only one item referring to “influencing decisions in the local area” proved problematic to interpret (Table 3). Carers considered potential difficulties with six questions (Table 3).

**Table 3:** Results of the cognitive debriefing

Item	Content	Concern by patients (Yes (n)/No)	Concern by carers (Yes/No)
Q1	Daily activities	Y (1)	Y
Q2	Social networks	Y (3)	Y
Q3	Losing sleep	Y (3)	Y
Q4	Enjoying recreation	N	Y
Q5	Suitable accommodation	N	Y
Q6	Neighbourhood safety	Y (1)	N
Q7	Potential for assault	Y (1)	N
Q8	Discrimination	N	N
Q9a	Influencing local decisions	Y (8)	N
Q9b	Freedom of expression	Y (1)	N
Q9c	Appreciating nature	N	N
Q9d	Respect and appreciation	N	N
Q9e	Love and support	N	Y
Q9f	Planning one’s life	N	N
Q9g	Imagination and creativity	N	N
Q9h	Access	N	N

Based on the qualitative analysis of the cognitive debriefing sessions, another six change suggestions were brought forward by the in-country investigator and three by the patients and/or carers (Table 1). Following careful linguistic and construct considerations including preserving the integrity of the instrument and focusing on common concerns, five of the suggested changes were implemented at this stage (Table 1).

Overall, 14 out of the 17 suggested changes were accepted (82%) resulting in a total of 12 changed phrases out of the 29 originally translated phrases (41%) in the final German version of the OxCAP-MH instrument (Table 1). The majority of the proposed changes (12 out of 17; 71%) referred to ambiguous wording and the possibility of different interpretations. One suggested change (6%) was due to a politically unacceptable expression, and one due to differences in political and social systems (6%). Three changes were suggested and implemented due to need for harmonisation for cross-country language differences between Austria and Germany (18%) (Table 1).

While professional translators were able to discuss changes that referred to pure linguistic issues, feedback from the in-country investigator, mental health specialists, and patients and carers resulted in modifications in wording mostly due to cultural and political differences between the countries (UK and Austria/Germany) as well as cross-country differences between Austria and Germany (Table 1). Response option 5 (R8a in Table 1) serves as an example of a change necessary due to the politically and culturally differential meaning of a word in the German language. The direct translation of “race” to “Rasse” had to be removed from the final German version, as “Rasse” is a term considered politically incorrect in the German language due to historical reasons. Changes to Question 9a were necessary due to relevant terminology differences between Austria and Germany. The word “local area” was initially translated as “Ortsgebiet” which was expected to be difficult to interpret for psychiatric patients according to the feedback from the in-country investigator and the mental health specialists from Germany. Consequently, the wording “Wohngebiet” (living area) was adopted in the final version of the questionnaire (Additional file 1).

Differences in cultural and political concepts between the UK and Germany/Austria were also reflected in the translation of Question 9a which asks about the ability to influence decisions affecting the local area, i.e. if patients have a ‘voice’ in their local area. At first glance this question seemed to be unclear to most participants in the pilot study (Table 3). Qualitative analysis revealed that this is likely to be due to the fact that the concept of participation in decision making at the community level seems more relevant in the Anglo-Saxon culture. Nevertheless, as many study participants were able to come up with accurate exemplifications, the question was left unchanged to protect the instrument’s integrity (Additional file 1).

As a result of cognitive debriefing, Question 9e was also modified. Originally phrased, the question asked if patients “find it easy to enjoy the love, care and support of [their] family and friends”. It was easy to understand but considered problematic by both carers and some patients, since the expression implies that someone has a supporting family to begin with. “*Family? In the old days, yes …*” (ID 007), “*If I actually got [support] from my family, I would gratefully accept it …*” (ID 008) and “*Sure, but to this end [support] would actually need to exist to begin with*” (ID 009) were three of the (translated) comments hinting a potential deficit experienced by people with mental health conditions in this respect and showing a very important dimension of quality of life in the context of mental disorders. In the final translation the linking word “and” was therefore replaced with “and/or” (“*Mir fällt es leicht, die Liebe, Fürsorge und Unterstützung meiner Familie und/oder Freunde anzunehmen und zu genießen*.”).

Contrary to the English language, the use of masculine and feminine forms is inherent in the German language, especially in written communication. The text of the questionnaire, however, only contained one word where this issue was relevant, i.e. ‘Freunde/Freundinnen’ meaning ‘friends’. In order to keep the questionnaire easy to read, only the male version was included in the translation (‘Freunde’). Neither carers nor patients noted or brought this issue up at the piloting stage and therefore no changes were deemed necessary.

Another characteristic of the German language is the use of “Du/Sie” forms. While the English “you” is used in both formal and informal communication, in German two variants are used for this personal pronoun: “Du” in informal and “Sie” in formal language. In line with German cultural standards, the “Sie” form was used throughout the questionnaire. No relevant concerns were raised.

Additional file 1 presents the full details of the translation process based on one sample item from the OxCAP-MH questionnaire.

## Discussion

This study describes the development of the German version of the OxCAP-MH well-being questionnaire that is both equivalent to the English source version and culturally acceptable and feasible to comprehend by German speaking patients. The development of the German version consisted of forward and back translations valid both for Austria and Germany. This was followed by cultural and linguistic validation through pilot testing with a sample of 12 German native speaking patients with mental health conditions in Austria. Incorporating this latter step allowed the development of a robust German translation of the OxCAP-MH with full formal linguistic and cultural adaptations.

The study followed a robust methodological design reflecting the principles of good practice for translation of patient-reported outcome measures adopted by ISPOR [19]. The aim was to create a valid German translation which is as close as possible to the original English source questionnaire. Feedback from the in-country investigator, mental health specialists, carers and cognitive debriefing with patients were particularly important to capture politically unacceptable expressions, cross-country language differences, and differences in cultural and political concepts. In total, 29 phrases comprising the OxCAP-MH instrument were translated from English into German. Changes in 31% of the phrases in the first translated version of the questionnaire (Version 1) were made based on the back translation review which involved two professional translators, the in-country investigator and mental health specialists from Germany. Final changes to the second translation were made in 17% of the phrases after obtaining feedback from patients and carers through pilot cognitive debriefing interviews. Through these linguistic and cultural adaptations, the final German version of OxCAP-MH is deemed equivalent to the original English questionnaire.

The pilot testing also allowed assessment of the patients’ perception and conceptual understanding of the German OxCAP-MH. The length of the full interviews ranged between 5 and 40 min. These differences in duration, however, were not linked to varying difficulties in understanding the questions but were driven by the patients’ interest in and willingness to discuss the broader meaning and concept of the questionnaire. The initial amount of time needed for going through all the 16 questions was around five minutes for each patient, indicating good feasibility of completion also for the German OxCAP-MH.

The German OxCAP-MH is now available for use free of charge for non-commercial use and is accessible via http://healtheconomics.meduniwien.ac.at/science-research/oxcap-mh/. Research on the full psychometric validation of the German questionnaire is ongoing in a large sample of patients with mental health conditions in Austria.

## Conclusions

This paper offers information about the linguistic and cultural transferability of the OxCAP-MH instrument, which measures broader well-being based on the capability approach and is considered a promising alternative for/addition to health-related quality of life measurement in mental health research, to a German speaking setting [9]. In the cognitive debriefing study, it was confirmed that the questions included in the instrument have personal relevance to the patients and the capability questionnaire represents important aspects of quality of life of people with mental health problems beside health. The study also demonstrates the necessity of conducting a formal, step-by-step translation and linguistic and cultural adaptation of a PROM instrument when developing different language versions. Pilot testing of the translation with patients and carers provided further valuable insights of the questionnaire’s content validity from their perspective. A larger scale study is, however, needed to establish the psychometric properties and validity of the German OxCAP-MH.

## Additional file

Additional file 1: Example of the translation process for one questionnaire item. (DOCX 16 kb)

## Abbreviations

OxCAP-MH: Oxford CAPabilities questionnaire-Mental Health
PROM: Patient-reported outcome measure
