# Supplementary material for: Cultural and linguistic transferability of the multi-dimensional OxCAP-MH capability instrument for outcome measurement in mental health: the German language version
Source: BMC Psychiatry. 2018 Jun 5;18:173. doi: 10.1186/s12888-018-1762-3 (PMC5987381; doi:10.1186/s12888-018-1762-3)
Supplement: Supplementary file 1 — Example of the translation process for one questionnaire item. (DOCX 16 kb) [file 12888_2018_1762_MOESM1_ESM.docx]

**Additional file 1: Table S1** Example of the translation process for one questionnaire item

| Q9a: Influencing local decisions | |
| --- | --- |
| English | I am able to influence decisions affecting my local area. |
| Reconciled German forward translation | Ich kann Entscheidungen beeinflussen, die sich auf mein Ortsgebiet auswirken. |
| First back translation | I can influence decisions, which have an effect on my locality. |
| Second back translation | I can have an influence on decisions that affect the area in which I live. |
| Reviewer | Discussed that the verb "am able to" was not suitable here as it referred to physical activity. |
| Developer | Comment that "Ortsgebiet" is very unusual in German and would not be easily understandable to psychiatric patients. Suggestion to use "Wohngebiet" or "Umgebung". |
| Lead translator | "Wohngebiet" suggested as an alternative. |
| New wording | Ich kann Entscheidungen beeinflussen, die sich auf mein Wohngebiet auswirken. |
| Back translation | I can influence decisions that affect the area in which I live. |
| Reviewer | Item to be worded as follows: Ich kann Entscheidungen beeinflussen, die sich auf mein Wohngebiet auswirken. |
| Developer | OK |
| Patients’ feedback | Some problems with understanding the question and connecting the context of the question to the real life. |
| Carers’ feedback | OK (based on example/explanation) |
| Researchers from Hamburg, Germany | Suggestion to use wording "nähere Umgebung" or "Umfeld" as it more accurately describes the "local area". |
| Developer | Suggestion to include the wording "nähere Umgebung" instead of "Wohngebiet" in the translated version. |
| Lead translator | Comment that the problem was not the comprehension of "local area (Wohngebiet)" but of "decisions (Entscheidungen)". Suggestion to use "gesellschaftspolitische Entscheidungen" (socio-political decisions). |
| Reviewer | Comment that adding ‘socio-political decisions’ would make the question clearer. |
| New wording | Ich kann gesellschaftspolitische Entscheidungen beeinflussen, die sich auf mein Wohngebiet auswirken. |
| Developer | Comment that "gesellschaftspolitische" is a difficult word to understand for mentally ill patients. Suggestion to not include it. |
| Final translation | Ich kann Entscheidungen beeinflussen, die sich auf mein Wohngebiet auswirken |
